# Inhibition of cell-associated HIV-1 by silver nanoparticles

**DOI:** 10.1186/1742-4690-9-S1-O1

**Published:** 2012-05-25

**Authors:** Dinesh K Singh, Humberto H Lara

**Affiliations:** 1 Winston Salem State University, Winston Salem Nc, USA

## Introduction

The glycoprotein gp120 and gp41 of HIV are the main targets for neutralizing antibodies (NABs). It appears that silver nanoparticles (AgNPs) also inhibit HIV-1 targeting same glycoproteins. In this study, we demonstrated that silver nanoparticles are efficient in neutralizing HIV-1 at non toxic concentrations. We also found an additive effect between the four NABs and AgNPs when combined against cell-associated HIV-1 infection in vitro.

## Materials and methods

The HIV-1IIIB virus, U373-MAGI-CXCR4CEM, HTLV-IIIB, Monoclonal antibody to HIV-1 gp41 (126-7), HIV-1 gp120 Antiserum (PB1 Sub 2), HIV-1 gp120 Antiserum (PB1), and HIV-1 gp120 Monoclonal Antibody (F425 B4e8) were obtained from the NIH AIDS Research and Reference Reagent Program, Division of AIDS, NIAID. The silver nanoparticles coated with 0.2 wt% PVP were obtained from Nanoamor, Houston, TX. Stock solutions and serial dilution of AGNPs were prepared in RPMI 1640 cell culture media. Cytotoxicity of AgNPs was ascertained in U373-MAGI-CXCR4CEM cells. The cell viability was assessed using a CellTiter-Glo® Luminescent Cell Viability Assay and Glomax Multidirection System (Promega). The neutralizing activity of AgNPs and NABs against HIVIIIB cell-free and cell associated virus was evaluated in an assay involving U373-MAGI-CXCR4CEM cells, AgNPs and NABs. Assessment of HIV-1 infection was performed with the Beta-Glo Assay System using Glomax Multidirection System (Promega). The percentage of residual infectivity after NABs, AgNPs, NABS+AgNPs, or media was calculated with respect to the positive control. The 50% inhibitory concentration (IC50) was defined according to the percentage of infectivity inhibition relative to the positive control. The inhibition data was statistically analyzed with the help of Wilcoxon rank-sum (Wilcoxon-Mann-Whitney test) test.

## Results

The four NABs used in the study inhibited HIV-1 cell free infection at a dose response manner. They were however largely ineffective against the cell-associated virus. AgNPs alone however were able to inhibit both cell free and cell associated virus infection at a dose dependent manner. AgNPs when mixed together with NABs significantly increased inhibition of Cell associated HIV-1.

## Conclusions

The addition of AgNPs to NABs has significantly increased the neutralizing potency of NABs in prevention of cell-associated HIV-1 transmission/infection.

